# The age patterns of severe malaria syndromes in sub-Saharan Africa across a range of transmission intensities and seasonality settings

**DOI:** 10.1186/1475-2875-9-282

**Published:** 2010-10-13

**Authors:** Arantxa Roca-Feltrer, Ilona Carneiro, Lucy Smith, Joanna RM Armstrong Schellenberg, Brian Greenwood, David Schellenberg

**Affiliations:** 1 Department of Disease Control, Faculty of Infectious & Tropical Disease, London School of Hygiene & Tropical Medicine, Keppel Street, London WC1E 7HT, UK

## Abstract

**Background:**

A greater understanding of the relationship between transmission intensity, seasonality and the age-pattern of malaria is needed to guide appropriate targeting of malaria interventions in different epidemiological settings.

**Methods:**

A systematic literature review identified studies which reported the age of paediatric hospital admissions with cerebral malaria (CM), severe malarial anaemia (SMA), or respiratory distress (RD). Study sites were categorized into a 3 × 2 matrix of *Plasmodium falciparum *transmission intensity and seasonality. Probability distributions were fitted by maximum likelihood methods, and best fitting models were used to estimate median ages and to represent graphically the age-pattern of each outcome for each transmission category in the matrix.

**Results:**

A shift in the burden of CM towards younger age groups was seen with increasing intensity of transmission, but this was not the case for SMA or RD. Sites with 'no marked seasonality' showed more evidence of skewed age-patterns compared to areas of 'marked seasonality' for all three severe malaria syndromes.

**Conclusions:**

Although the peak age of CM will increase as transmission intensity decreases in Africa, more than 75% of all paediatric hospital admissions of severe malaria are likely to remain in under five year olds in most epidemiological settings.

## Background

The relationship between the age-pattern of severe malaria and transmission intensity has been studied widely. Several studies conducted in the 1990s reported a 'peak shift' phenomenon - the peak incidence of hospital admissions with severe malaria occurring at a younger age in areas of high transmission intensity compared to areas of lower transmission intensity[1-5] and this finding has been confirmed by a recent pooled analysis[6].

Severe malaria can present clinically in several different ways. The syndromes encountered most frequently in African children are severe malaria anaemia (SMA), cerebral malaria (CM) or respiratory distress (RD)[1]. Previous studies of the age pattern of individual severe malaria syndromes and its relationship to transmission intensity have shown a shift in the peak of admissions with SMA or CM [7,8] toward younger ages with increasing transmission intensity, but this has not been apparent for RD[8]. In addition, it has been shown that the mean age of hospital admissions with SMA is always lower than that of admissions with CM, regardless of transmission intensity. As a result, it is widely believed that SMA will dominate the clinical picture in areas of high transmission, whilst CM should become relatively more important in areas with a lower transmission intensity[9]. However, not all studies have observed such a relationship: in Ifakara, Tanzania an area which previously had intense malaria transmission, a similar incidence of hospital admissions with CM or SMA was reported in under-five year olds[10].

There are likely to be several reasons for the inconsistencies observed between studies, including a lack of standardized definitions of transmission intensity, difficulty in allocating patients to specific clinical syndromes, the use of different age groupings and age ranges across studies, and the small number of studies between which comparisons can be made. Although there has been an attempt to overcome these limitations by looking at this relationship across a wider range of transmission intensities and using standard clinical definitions [11], it still remains unclear whether the 'peak shift' phenomenon occurs for all severe malaria syndromes. To date, no studies have included the role of malaria seasonality in the analysis of the relationship between the age-pattern of severe malaria syndromes and transmission intensity.

This paper presents a pooled analysis of existing data that describes the age-pattern of severe malaria syndromes across a wide range of transmission intensities and seasonality settings in sub-Saharan Africa. The methodology used overcomes the difficulty of combining studies that report different age-groupings and age-ranges. This has enabled inclusion of data from a variety of transmission settings, including data from studies conducted outside established research sites.

## Methods

### Literature review

To identify relevant data on the age-pattern of severe malaria syndromes, a series of systematic literature reviews were undertaken between 2005 and 2006 which are reported in more detail elsewhere[6]. PubMed and CAB Abstracts (BIDS) electronic databases were searched using the following terms: "malaria (with a major focus on epidemiology, complications, mortality, prevention and control, and transmission) OR *Plasmodium falciparum *OR *Plasmodium vivax*" AND "morbidity (incidence or prevalence)" OR "fever" OR "severe malaria" or "cerebral malaria" OR "neurological" OR "an(a)emia". In addition, searches of the WHO library (WHOLIS)[12], and the grey literature (SIGLE) database[13] were undertaken. References were also identified by conducting key author searches and checking cross-references from the bibliographies of relevant papers. Additional data sources, such as the Severe Malaria in African Children (SMAC) clinical trials network, were also contacted to obtain information on individual hospital admissions.

Only studies from countries endemic for *P. falciparum *and reporting age-breakdown data of hospital admissions with the main severe malaria syndromes in children up to 15 years were included. CM, SMA and RD definitions varied between studies and did not necessarily fulfill WHO definitions [14-16] as authors tended to adapt WHO definitions according to the local epidemiology. However, all severe malaria cases included in these analyses were parasitologically confirmed for *P. falciparum*. In addition, "impaired consciousness" or "unrousable coma" were necessary for inclusion as a CM case, all SMA cases included had either Hb ≤ 5.0 g/dL or a PCV ≤ 15%, and all RD cases had "acidosis" or "deep breathing".

### Categorizing studies into a matrix of transmission intensity and seasonality

Currently, the preferred measure for assessing malaria endemicity is the annualized entomological inoculation rate (EIR) defined as the number of malaria infective bites per person per year. However, as measuring EIR is resource-intensive, EIR data across sub-Saharan Africa is scarce. Beier *et al *[17] reported a linear relationship between malaria prevalence and the logarithm of the annual EIR, justifying the use of parasite prevalence as a marker of transmission intensity in areas where EIR data is not available. Therefore, to identify relevant literature on transmission intensity, two systematic literature reviews of studies measuring EIR and/or parasite prevalence were also undertaken as described previously[6].

Studies were categorized into a 3 × 2 matrix of malaria transmission intensity (EIR: < 10, 10-100, > 100 infectious bites per person per year (pppy)) and seasonality ('marked seasonality', 'no marked seasonality'). Where available, geo-referenced estimates of EIR meeting minimum quality criteria were matched to each study. For sites where no suitable EIR data were available, cut-offs of parasite prevalence in under-five year olds (< 25%, 25-60% and > 60%) were used to categorize sites into low, medium, or high transmission intensity. This categorization was based on an independent analysis of the relationship of EIR and malaria prevalence[18] that was consistent with the previously described log-linear relationship between EIR and parasite prevalence[17,19].

There is no standard definition of seasonality of malaria transmission. Studies where an assessment of seasonality could be made were categorized into 'marked seasonality' (those with ≥ 75% of episodes concentrated in ≤ 6 months of the year) or 'no marked seasonality' as described in detail previously[20]. Sites in which an assessment of seasonality could not be made were categorized according to the Mapping Malaria in Africa (MARA) database[21]. Expert opinion was sought for two sites (Ilorin and Calabar, Nigeria) where no data on transmission intensity were available. For each severe malaria syndrome results were allocated to one of six cells of a transmission intensity-seasonality matrix: low, medium, high transmission, and 'marked' or 'no marked seasonality' (Table 1).

**Table 1:** Number of sites(studies) included in the main analyses for each cell of the transmission intensity-seasonality matrix.

Transmission intensity (in pppy)	Marked seasonality			No marked seasonality		
		
	CM	SMA	RD	CM	SMA	RD
**< 10**	3(8)	3(7)	-	2(2)	-	2(3)

**10-100**	3(4)	4(4)	4(6)	7(11)	10(15)	1(1)

**> 100**	2(2)	1(1)	1(1)	-	2(2)	-

Cerebral malaria = CM; severe malarial anaemia = SMA; and respiratory distress = RD

### Data analysis

For each syndrome, data from sites in the same cell of the transmission matrix were analysed together. Five probability distributions (Gamma, Exponential, Weibull, Log-logistic, Log-normal) were fitted to data using a user defined module ('intcens' command) in Stata 10 (StataCorp. 2007. Stata Statistical Software: Release 10. College Station, TX: StataCorp LP). The 'intcens' command fits various distributions by maximum likelihood to a non-negative outcome (e.g. cerebral malaria cases). As studies identified through the literature review differed in age-groupings and age-ranges, interval-censored models were used to account for the fact that events between two ages were not recorded exactly in the dataset, and that events were recorded up to different maximum ages in each study. The proportion of each syndrome by month of age between 0 and10 years was calculated for each study site. The distributions with the lowest AIC (Akaike Information Criterion) value were identified as the best fitting [22]. Studies that only included children aged less than 4 years old were excluded from the analyses to avoid skewing the age-distributions to younger age groups. Sites with less than 40 observations for a given outcome were also excluded from the analysis to avoid spurious percentage age-distributions. However, a sensitivity analysis was performed, including all available studies regardless of their size and a comparison with the main analysis is presented. The median and inter-quartile range (IQR) of age was calculated from the best-fitting distribution for each outcome and each cell of the transmission intensity-seasonality matrix.

## Results

A total of 27, 29 and 11 studies in 26 distinct sites in 15 countries of sub-Saharan Africa were included in the main analysis of CM, SMA, and RD respectively (Tables 1 and additional file 1). The largest studies reported the full paediatric age-range (< 15 years) for malaria admissions (see additional file 1). Age-breakdown data for CM were not identified in areas with 'no marked seasonality' and high transmission intensity. No studies were available for predicting age-patterns of SMA in 'no marked seasonality' and low intensity settings. Predictions for RD could not be made for two transmission settings ('marked seasonality' and high intensity and 'no marked seasonality' and low intensity) as no studies reporting age-breakdown data were available in these categories. For each outcome and for each epidemiological setting, different probability distributions gave the best fit, across different epidemiological settings (Table 2).

**Table 2:** Best fitted distributions, Akaike Information Criterion (AICs), and estimated median ages (IQR) ^∫ ^for each severe outcome and each cell of the transmission intensity-seasonality matrix

Transmission Matrix category	Cerebral malaria			Severe Malarial Anaemia			Respiratory Distress		
			
	Best fitted model	AIC	Median age (IQR)	Best fitted model	AIC	Median age (IQR)	Best fitted model	AIC	Median age (IQR)
**Marked Seasonality:**									

**EIR < 10 pppy**	Log-Logistic	13272	49 (31-72)	Log-Logistic	14146	27 (16-44)	Log-Logistic	7501	34 (19-57)

**EIR 10-100 pppy**	Gamma	4420	42 (25-64)	Log-Logistic	4799	25 (15-40)	Log-Normal	1161	36 (20-61)^Ω^

**EIR > 100 pppy**	Log-Normal	497	34 (19-57)	Weibull	259	30 (15-52)	-	-	-

**No marked seasonality**									

**EIR < 10 pppy**	Log-Normal	672	41 (26-63)	-	-	-	-	-	-

**EIR 10-100 pppy**	Log-Logistic	20714	29 (18-47)	Log-Normal	47782	17 (10-29)	Log-Normal	20334	21 (12-36)

**EIR > 100 pppy**	-	-	-	Log-Logistic	3697	15 (9-25)	Log-Normal	523	23 (15-37)^¥^

^∫^Same median age (IQR) results were obtained in the sensitivity analyses except when otherwise indicated

^Ω^Median age (IQR) obtained from sensitivity analyses: 36 (20-62)

^¥^Median age (IQR) obtained from sensitivity analyses: 22 (13-36)

**Figure 1:**
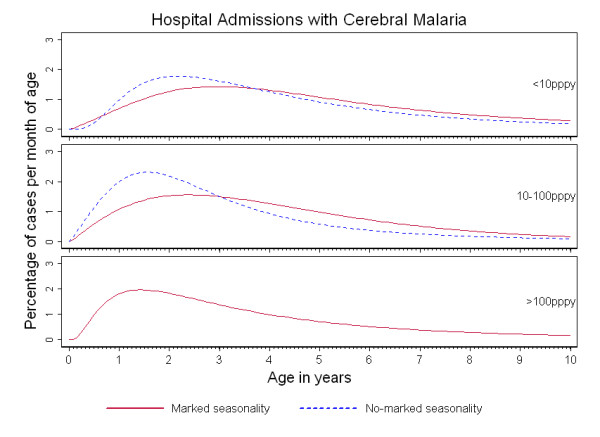
**Age-patterns of cerebral malaria in Sub-Saharan Africa**. Percentage of cerebral malaria admissions per month of age in children under ten years of age, by transmission intensity (TI) and seasonality of malaria transmission.

**Figure 2:**
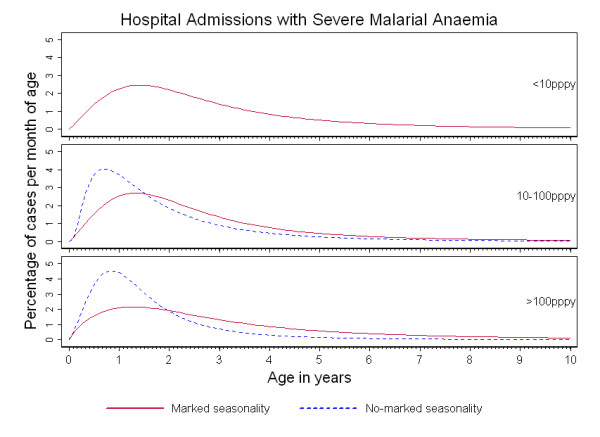
**Age-patterns of severe malarial anaemia in Sub-Saharan Africa**. Percentage of severe malarial anaemia admissions per month of age in children under 10 years of age, by transmission intensity (TI) and seasonality of malaria transmission.

**Figure 3:**
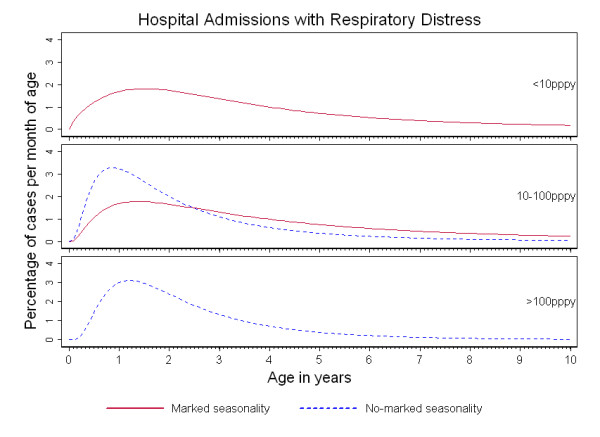
**Age-patterns of respiratory distress in Sub-Saharan Africa**. Percentage of respiratory distress admissions per month of age in children under 10 years of age, by transmission intensity (TI) and seasonality of malaria transmission.

Figures 1, 2 and 3 show the percentage distribution of each severe malaria syndrome by age for children under 10 years, such that the integral of the curve is equal to 100% of expected cases. As shown in these figures, the age-pattern of each specific syndrome varied by seasonality: for a given transmission intensity, a shift of the peak age to younger children was observed in 'no marked seasonality' settings compared to 'marked seasonality' settings.

Cerebral malaria admissions were concentrated in under-five year olds with more than 75% of cases in this age group in all 'no marked seasonality' settings. In areas of 'marked seasonality', CM cases were more evenly distributed across childhood, except for settings of high transmission intensity. In addition, a pronounced shift towards younger age groups was observed with increasing transmission intensity, both in areas of 'no marked seasonality' and 'marked seasonality' (Figure 1). In areas of 'marked seasonality', the median age of cases of CM decreased from 49 months (IQR: 31, 72) in settings of low intensity transmission to 34 months (IQR: 19, 57) in high intensity transmission settings. For settings with 'no marked seasonality', median age decreased from 41 months (IQR: 26, 63) in low transmission settings to 29 months (IQR: 18, 47) in medium transmission intensity settings (Table 2).

Younger peak ages were seen also for hospital admissions with SMA in 'no marked seasonality' compared to 'marked seasonality' settings and for any transmission intensity setting (Figure 2). However, for a given seasonality category, no marked differences were observed by transmission intensity, resulting in similar predicted median ages in each of the seasonality categories The median age of cases of SMA did not decrease with increasing transmission intensity) (Table 2).

In the case of admissions with RD, a comparison of age-patterns by type of seasonality could be made only in settings of medium transmission due to the small number of studies available to make predictions. However, younger peak ages were consistently found in 'no marked seasonality' compared to 'marked seasonality' settings, as seen for the other severe malaria syndromes. Again, no apparent shift towards younger ages was seen with increasing transmission regardless of seasonality. Similar median ages were predicted in 'marked seasonality' low transmission settings (34 months (IQR: 19, 57) and in 'marked seasonality' medium transmission settings (36 months (IQR: 20, 61). Similarly in 'no marked seasonality' settings median predicted ages in medium intensity and in high transmission intensity settings were 21 months (IQR: 12, 36) and 23 months (IQR: 15, 37) respectively.

**Figure 4:**
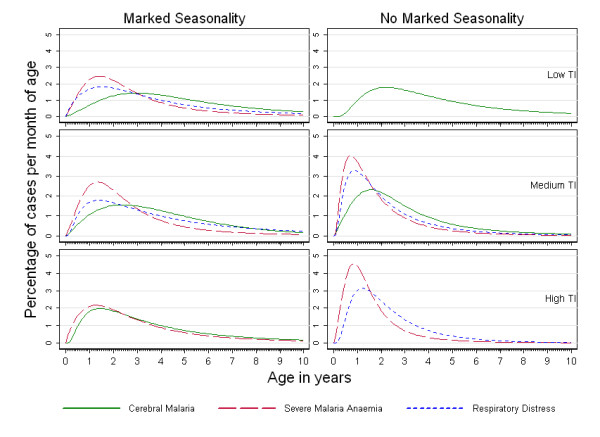
**Age-patterns of cerebral malaria, severe malarial anaemia, and respiratory distress in Sub-Saharan Africa**. Percentage of cerebral malaria, severe malarial anaemia, and respiratory distress admissions per month of age in children under 10 years of age, for each cell of the transmission intensity-seasonality matrix.

Although all specific severe malaria syndromes were concentrated in under-fives, the predicted age-patterns of CM compared to SMA were found to be different: hospital admissions with SMA were more concentrated in younger children than cases of CM. This finding was consistent within each transmission intensity category and by type of seasonality (Figure 4).

Sensitivity analyses allowed inclusion of 13 additional studies (see additional file 1). The same best fitted models were obtained for all epidemiological settings, and very similar median ages were predicted after including all available studies regardless of study size (Table 2). Inclusion of studies with a small size had little effect on the estimated age-pattern of any severe malaria syndrome by transmission intensity or seasonality.

## Discussion

A better understanding of the relationship between transmission intensity, seasonality and the age-pattern of malaria is needed to target interventions in different epidemiological settings. Here we present the most comprehensive overview of the age-pattern of severe malaria syndromes undertaken to date including data from a wide variety of epidemiological settings. The inclusion of all identified studies with age-breakdown data in the predictions was possible by using 'interval-censored analysis'[23] for fitting statistical distributions to age-breakdown data. This method proved to be a valuable approach for overcoming the difficulty of combining studies that differed in age-groupings and age-ranges.

Our findings confirm previous observations of a shift in the burden of CM towards younger age groups with increasing transmission intensity. However, no such shift was apparent for SMA or RD. There are many causes of severe anaemia in young children in addition to malaria, including malnutrition and HIV. The complex aetiology of anaemia is likely to vary with age, and might mask an underlying relationship between SMA and transmission intensity. The lack of evidence of an age-dependent relationship between RD and transmission intensity might be explained by the small number of epidemiological settings available to make predictions, but also by the fact that children admitted with RD frequently present with associated SMA or CM [24] and may have been categorized as cases of SMA or CM, but not RD; it is uncommon for RD to be the single feature of severe malaria. Unfortunately, information on the extent to which all three severe malaria syndromes overlapped, was not available as data were provided by age and not individual, therefore each syndrome was dealt with independently in the analyses. Sites with 'no marked seasonality' showed more skewed age-patterns compared to areas of 'marked seasonality' for all severe malaria syndromes. In the case of CM, the effect of seasonality appeared to dampen the impact of transmission intensity on the observed age-patterns. Even if the intensity of transmission is similar in two different areas over a one-year period, children who live in an area where exposure to malaria is restricted to only a few months a year acquire protective immunity more slowly than children who live in an area where exposure occurs all year-round, presumably because they do not have the boosting effect of repeated exposure from perennial transmission. Similar observations were made in a parallel study that described the age-profiles of clinical malaria, hospital admissions with malaria and malaria-diagnosed mortality in a wide range of transmission settings using the same methodology[6]. Seasonality plays an important role in shaping the age-pattern of severe malaria and should not be neglected when developing malaria control programmes[20].

A number of biases and limitations may have distorted our predictions. Firstly, our literature review identified geographical clustering in the distribution of studies, reflecting the bias in malaria research towards established research centres and urban areas. Hospitals are generally located in urban or semi-urban areas and this may have introduced some misclassification when assigning a transmission intensity category to a given site. Children living in high transmission rural areas attending these hospitals might have been misclassified and this could have distorted the predicted age-patterns presented here. As hospital catchment areas vary by site it is difficult to estimate the direction and magnitude of this potential bias. Secondly, there was a paucity of data from some epidemiological settings such as 'no marked seasonality' and low transmission. Lack of data for these areas might be explained by the fact that malaria research, on severe syndromes especially, is biased towards medium to high transmission intensity areas to obtain sufficient numbers of cases[6]. Further information from this epidemiological setting is sorely needed, particularly as a higher proportion of the population in sub-Saharan Africa lives in these areas than previously thought[25]. Thirdly, the predicted age-patterns in these analyses are based on hospital admissions for severe malaria which may not match age-patterns of severe malaria in the community. Failure to present to hospital may be more likely for infants than for older children. This difference in the presentation to hospital by age may vary for specific severe malaria syndromes, for example SMA may be relatively unrecognized by the carer in comparison with CM[26]. However, the features of CM may be perceived as being due to witchcraft, and traditional healers are more likely to be consulted for this form of severe malaria than for SMA[27]. In short, the proportion of cases that present to hospital is not known and may vary by syndrome and age, so our predictions may not represent the age-pattern of severe malaria cases in the community. Finally, some misclassification may have occurred when allocating sites to a cell of the transmission intensity-seasonality matrix. Although an effort was made to match EIR data for each site by season and year, this was challenging due to lack of available site-specific EIR data. Whenever possible, expert knowledge was sought to support the categorization. The same applies to allocation of sites to type of seasonality. However, sensitivity analyses of the definition used to categorize studies by seasonality showed consistent patterns for different malaria outcomes and across several years[20]. Lastly, it was not possible to perform sensitivity analyses to explore the potential impact of different definitions on the age-pattern of specific severe syndromes due to lack of available information as authors not always specified the detailed diagnostic criteria. Where studies used more stringent definitions to define a specific syndrome, this would have resulted in fewer cases fulfilling the definition, but it unlikely to have affected the proportion of cases in each age-group.

The age pattern of severe disease within each site should, therefore, have been maintained despite a variation in for example, threshold parasite densities for defining SMA between sites.

The results of this study are consistent with previous studies [1-5,7,28,29] that have reported that the median age of patients with SMA is lower than that of patients with CM. However, this study is the first to have taken seasonality into account. The underlying biological reasons for this differential age effect remain unclear. Hypotheses include physiological immaturity in receptors involved in the sequestration of infected red cells making infants less susceptible to CM, and the possibility that CM is caused by rare parasite variants that simply by chance are encountered at an older age[2,5]. In the context of the move towards malaria elimination [30,31], our findings suggest that the peak age of CM will increase as control interventions decrease transmission intensity, whereas little change would be predicted in the age distribution of patients with either SMA or RD. If this is the case, an increase in the relative importance of CM *vs*. SMA as the intensity of malaria transmission declines would be expected, a finding that has been recently been reported in the literature[9]. This prediction assumes that areas where the level of transmission has been reduced through control are identical to those where this level of transmission has occurred through other means. There is growing evidence suggesting that the age-patterns of malaria in sites experiencing a transition may take some time to stabilize and show age-patterns corresponding to lower transmission levels[9,11]. Nevertheless, the presented findings suggest that, for most epidemiological settings, more than 75% of all paediatric hospital admissions with severe malaria will occur in under five year olds. However, as predictions were restricted to children under the age of 10 years, it is difficult to estimate what contribution to the overall burden of severe malaria in the community will be made by children under five years old as the incidence of malaria declines to a low level. In these circumstances, teenagers (10 to 15 years old) may account for a substantial proportion of cases of paediatric severe malaria as seen currently in many areas outside Africa.

Deciding which age-group malaria interventions should target is complicated by the fact that the age pattern of malaria deaths has been shown to be different from the age pattern of non-severe malaria and severe malaria[6,32]. As scaling-up of malaria intervention continues it will be crucial to monitor not only changes in the burden of malaria but also changes in the age-pattern of malaria syndromes to better understand the impact of reducing the intensity of malaria transmission. Monitoring changes in the age-pattern of malaria will help us to identify which age-targeted interventions may be most appropriate in each epidemiological setting, as sites may experience transmission intensity and seasonality transitions as scaling up of malaria control interventions continues.

## Competing interests

The authors declare that they have no competing interests.

## Authors' contributions

ARF, IC, JAS, BG, and DS conceived and designed the analyses. ARF, IC and LS collected the data. ARF and IC analyzed the data. ARF drafted the manuscript, and all authors made significant contributions to the manuscript and critically reviewed the final version.

## Funding

This project was funded by The Bill and Melinda Gates Foundation (Grant ID 33679; http://www.gatesfoundation.org through the Intermittent Preventive Treatment in Infants (IPTi) consortium. I.C. is also funded by the Department for International Development, United Kingdom http://www.dfid.gov.uk through the TARGETS Communicable Disease Consortium (Grant ID HD205). The funders had no role in study design, data collection and analysis, decision to publish, or preparation of the manuscript.

## Supplementary Material

Additional file 1 **Characteristics of studies included in the analyses (sorted by country and site) by syndrome and by transmission intensity and seasonality categories**. Click here for file

## References

[B1] Marsh K Forster D Waruiru C Mwangi I Winstanley M Marsh V Newton C Winstanley P Warn P Peshu N Pasvol G Snow R Indicators of life-threatening malaria in African children N Engl J Med 1995 332 1399 1404 10.1056/NEJM199505253322102 7723795

[B2] Snow RW Bastos de Azevedo I Lowe BS Kabiru EW Nevill CG Mwankusye S Kassiga G Marsh K Teuscher T Severe childhood malaria in two areas of markedly different falciparum transmission in east Africa Acta Trop 1994 57 289 300 10.1016/0001-706X(94)90074-4 7810385

[B3] Imbert P Sartelet I Rogier C Ka S Baujat G Candito D Severe malaria among children in a low seasonal transmission area, Dakar, Senegal: influence of age on clinical presentation Trans R Soc Trop Med Hyg 1997 91 22 24 10.1016/S0035-9203(97)90380-1 9093619

[B4] Modiano D Sirima BS Sawadogo A Sanou I Pare J Konate A Pagnoni F Severe malaria in Burkina Faso: influence of age and transmission level on clinical presentation Am J Trop Med Hyg 1998 59 539 542 9790426 10.4269/ajtmh.1998.59.539

[B5] Marsh K Snow RW Malaria transmission and morbidity Parassitologia 1999 41 241 246 10697862

[B6] Carneiro I Roca-Feltrer A Griffin JT Smith L Tanner M Schellenberg J Greenwood B Schellenberg D Age-patterns of malaria vary with severity, transmission intensity and seasonality in sub-Saharan Africa: A systematic review and pooled analysis PLoS One 2010 5 e8988 10.1371/journal.pone.0008988 20126547 PMC2813874

[B7] Reyburn H Mbatia R Drakeley C Bruce J Carneiro I Olomi R Cox J Nkya WM Lemnge M Greenwood BM Riley EM Association of transmission intensity and age with clinical manifestations and case fatality of severe *Plasmodium falciparum *malaria JAMA 2005 293 1461 1470 10.1001/jama.293.12.1461 15784869

[B8] Idro R Aloyo J Mayende L Bitarakwate E John CC Severe malaria in children in areas with low, moderate and high transmission intensity in Uganda Trop Med Int Health 2006 11 115 124 10.1111/j.1365-3156.2005.01518.x 16398762

[B9] O'Meara WP Bejon P Mwangi TW Okiro EA Peshu N Snow RW Newton CRJC Marsh K Effect of a fall in malaria transmission on morbidity and mortality in Kilifi, Kenya Lancet 2008 372 1555 1562 10.1016/S0140-6736(08)61655-4 18984188 PMC2607008

[B10] Schellenberg D Menendez C Kahigwa E Font F Galindo C Acosta C Schellenberg JA Aponte JJ Kimario J Urassa H Mshinda H Tanner M Alonso P African children with malaria in an area of intense *Plasmodium falciparum *transmission: features on admission to the hospital and risk factors for death Am J Trop Med Hyg 1999 61 431 438 10497986 10.4269/ajtmh.1999.61.431

[B11] Okiro EA Al-Taiar A Reyburn H Idro R Berkley JA Snow RW Age patterns of severe paediatric malaria and their relationship to *Plasmodium falciparum *transmission intensity Malar J 2009 8 4 10.1186/1475-2875-8-4 19128453 PMC2630996

[B12] The WHO Publication Library (WHOLIS) http://www.who.int/library/databases/en/Accessed October 21, 2009

[B13] The SIGLE grey literature database http://opensigle.inist.fr/Accessed October 21, 2009

[B14] Warrell DA Gilles HM Essential malariology 2002 London: Arnold

[B15] WHO WHO Expert Committee on Malaria, Twentieth Report. Geneva, Switzerland WHO Tech Rep Ser 2000 3

[B16] WHO Severe falciparum malaria. Communicable Diseases Cluster Trans R Soc Trop Med Hyg 2000 94 1 S1 90 10.1016/S0035-9203(00)90413-9 11103309

[B17] Beier JC Killeen GF Githure JI Short report: entomologic inoculation rates and *Plasmodium falciparum *malaria prevalence in Africa Am J Trop Med Hyg 1999 61 109 113 10432066 10.4269/ajtmh.1999.61.109

[B18] Roca-Feltrer A Estimating The Burden And The Age Pattern Of Malaria Morbidity In Sub-Saharan Africa In Under-Fives 2008 University of London

[B19] Hay SI Rogers DJ Toomer JF Snow RW Annual *Plasmodium falciparum *entomological inoculation rates (EIR) across Africa: literature survey, Internet access and review Trans R Soc Trop Med Hyg 2000 94 113 127 10.1016/S0035-9203(00)90246-3 10897348 PMC3204456

[B20] Roca-Feltrer A Armstrong Schellenberg J Smith L Carneiro I A simple method for defining malaria seasonality Malar J 2009 8 276 10.1186/1475-2875-8-276 19958535 PMC3224898

[B21] Mapping Malaria Risk in Africa (MARA) Collaboration Towards an atlas of malaria risk in Africa. First technical report of the MARA/ARMA Collaboration 1998 Albany Print: Durban, South Africa http://www.mara.org.za/trview_e.htm (Accessed September 27, 2007)

[B22] Anderson DR Burnham KP Model selection and multimodel inference: a practical information-theoretic approach 2002 2 Springer

[B23] Farrington C Gay N Interval-censored survival data with informative examination times: parametric models and approximate inference Stat Med 1999 18 1235 1248 10.1002/(SICI)1097-0258(19990530)18:10<1235::AID-SIM120>3.0.CO;2-R 10363342

[B24] Roca-Feltrer A Carneiro I Armstrong Schellenberg J Estimates of the burden of malaria morbidity in Africa in children under the age of 5 years Trop Med Int Health 2008 13 1 13 10.1111/j.1365-3156.2008.02076.x 18363586

[B25] Malaria Atlas Project http://www.map.ox.ac.uk Accessed February 10, 2010

[B26] Schellenberg D Schellenberg JR Mushi A Savigny D Mgalula L Mbuya C Victora CG The silent burden of anaemia in Tanzanian children: a community-based study Bull World Health Organ 2003 81 581 590 Epub 2003 Oct 2014 14576890 PMC2572520

[B27] Baume C Helitzer D Kachur SP Patterns of care for childhood malaria in Zambia Soc Sci Med 2000 51 1491 1503 10.1016/S0277-9536(00)00049-6 11077952

[B28] Brewster DR Kwiatkowski D White NJ Neurological sequelae of cerebral malaria in children Lancet 1990 336 1039 1043 10.1016/0140-6736(90)92498-7 1977027

[B29] Gupta S Hill AV Kwiatkowski D Greenwood AM Greenwood BM Day KP Parasite virulence and disease patterns in *Plasmodium falciparum *malaria Proc Natl Acad Sci USA 1994 91 3715 3719 10.1073/pnas.91.9.3715 8170975 PMC43652

[B30] Bill and Melinda Gates Foundation Malaria Forum - Day 2, 17 October 2007 [Transcript] http://www.gatesfoundation.org/speeches-commentary/Pages/bill-gates-malaria-forum.aspx Accessed July 14, 2008

[B31] Roberts L Enserink M Did they really say...eradication? Science 2007 318 1544 1545 10.1126/science.318.5856.1544 18063766

[B32] Carneiro I Smith L Ross A Roca-Feltrer A Greenwood B Schellenberg J Smith T Schellenberg D Intermittent preventive treatment in infants: a decision-support tool for implementation Bull World Health Organ in press 21076561 10.2471/BLT.09.072397 PMC2971505

