# Supplementary material for: The age patterns of severe malaria syndromes in sub-Saharan Africa across a range of transmission intensities and seasonality settings
Source: Malar J. 2010 Oct 13;9:282. doi: 10.1186/1475-2875-9-282 (PMC2992028; doi:10.1186/1475-2875-9-282)
Supplement: Additional file 1 — Characteristics of studies included in the analyses (sorted by country and site) by syndrome and by transmission intensity and seasonality categories. [file 1475-2875-9-282-S1.DOCX]

**Additional File 1.** Characteristics of studies included in the analyses (sorted by country and site) by syndrome and by transmission intensity and seasonality categories.

| **Transmission matrix** | **Site (Country)** | **Reference** | **Study Period** | **Number of admissions** | **Age-range (years)** |
| --- | --- | --- | --- | --- | --- |
| **Cerebral malaria** |  |  |  |  |  |
| No marked seasonality and EIR<10pppy | Accra (Ghana)  Kampala (Uganda) | [1]  [2] | Jul to Oct 1979  Jul to Dec 2000 | 43  100 | 0-12  0-12 |
| No marked seasonality and EIR 10-100pppy | Brazzaville (Congo)  Lambarene (Gabon)  Libreville (Gabon)  Kumasi (Ghana)  Kumasi (Ghana)  Kilifi (Kenya)  Kilifi (Kenya)  Kilifi (Kenya)  Kilifi (Kenya)  Blantyre (Malawi)  Calabar (Nigeria) | [3]  [4]  [4]  [5]  [4]  [6]  [7]  [8]  [4]  [4]  [9] | Jan 1988 to Jun 1989  Dec 2000 to Apr 2005  Dec 2000 to Apr 2005  1993  Dec 2000 to Apr 2005  May 1989 to Apr 1992  Jul 1993 to Sep 1994  Sep 1999 to Dec 2000  Dec 2000 to Apr 2005  Dec 2000 to Apr 2005  Jan 1986 to Jun 1988 | 120  94  204  189  839  131  65  324  727  300  75 | 0-14  0-14  0-14  0-13  0-14  0-12  0-13  0-13  0-14  0-14  0-15 |
| *Additional studies for sensitivity analysis^∫^* | HJH-Kinshasa (DRC)  PHK-Kinshasa (DRC) Libreville(Gabon)  Manhica (Mozambique)  Moshi (Tanzania) | [10]  [10]  [11]  [12]  [13] | Sep to Dec 2004  Sep to Dec 2004  Jan to Dec 1992  Jun 2003 to May 2005  Feb 2002 to Feb 2003 | 13  12  12  24  24 | 0-12  0-12  0-15  0-15  0-13 |
| No marked seasonality and EIR >100pppy | - | - | - | - | - |
| Marked seasonality and <10pppy | Banjul (Gambia, The)  Banjul (Gambia, The)  Banjul (Gambia, The)  Banjul (Gambia, The)  Sibanor (Gambia, The)  Dakar (Senegal)  Dakar (Senegal)  Dakar (Senegal) | [14]  [15]  a  [4]  [15]  [16]  [17]  [18] | Sep to Dec 1988  Aug 1992 to Nov 1994  Dec 1996 to Nov 2000  Dec 2000 to Apr 2005  Aug 1992 to Nov 1994  Jan 1990 to Feb 1996  Oct 1997 to Mar 1999  Jul 1998 to Dec 1999 | 392  344  512  283  235  71  80  189 | 0-9  0-9  0-15  0-14  0-9  0-15  0-14  0-15 |
| *Additional studies for sensitivity analysis^∫^* | Geradif (Sudan) | [19] | Oct 2000 to Dec 2001 | 14 | 0-14 |
| Marked seasonality and 10-100pppy | Ouagadougou (Burkina Faso)  Maputo (Mozambique)  Maputo (Mozambique)  Macha (Zambia) | [20]  [21]  [22]  b | Jun to Oct 1993/1994  Feb to Jun 1990  Feb 1995 to Apr 1997  Jan 2003 to Dec 2004 | 660  82  178  75 | 0-15  0-7  0-6  0-5 |
| Marked seasonality and >100pppy | Sorou and Nayala (Burkina Faso)  Mnero (Tanzania) | [20]  [23] | Jun to Oct 1996  1979-1981 | 86  66 | 0-15  0-15 |
| **Severe malarial anaemia** | | | | | |
| No marked seasonality and EIR<10pppy | - | - | - | - | - |
| No marked seasonality and EIR 10-100pppy | HJH-Kinshasa (DRC)  PHK-Kinshasa (DRC)  Lambarene (Gabon)  Libreville (Gabon)  Libreville (Gabon)  Kumasi (Ghana)  Kilifi (Kenya)  Kilifi (Kenya)  Kilifi (Kenya)  Blantyre (Malawi)  Blantyre (Malawi)  Mangochi (Malawi)  Manhica (Mozambique)  Ifakara (Tanzania)  Moshi (Tanzania) | [10]  [10]  [4]  [11]  [4]  [4]  [6]  [8]  [4]  [24]  [4]  [24]  [12]  [25]  [13] | Sep to Dec 2004  Sep to Dec 2004  Dec 2000 to Apr 2005  Jan to Dec 1992  Dec 2000 to Apr 2005  Dec 2000 to Apr 2005  May 1989 to Apr 1992  Sep 1999 to Dec 2000  Dec 2000 to Apr 2005  Mar 1990 to Feb 1991  Dec 2000 to Apr 2005  Mar 1990 to Feb 1991  Jun 2003 to May 2005  Jan to Dec 1995/2000  Feb 2002 to Feb 2003 | 62  44  306  70  572  2167  137  115  1071  552  628  484  187  473  146 | 0-12  0-12  0-14  0-15  0-14  0-14  0-12  0-13  0-14  0-15  0-14  0-15  0-15  0-15  0-13 |
| *Additional studies for sensitivity analysis^∫^* | Ilorin (Nigeria) | [26] | Jan to Dec 1998 | 10 | 0-15 |
| No marked seasonality and EIR >100pppy | Tamale (Ghana)  Teule (Tanzania) | [27]  [13] | Aug 2002 to Nov 2002  Feb 2002 to Feb 2003 | 169  317 | 0-9  0-13 |
| Marked seasonality and <10pppy | Banjul (Gambia, The)  Banjul (Gambia, The)  Banjul (Gambia, The)  Dakar (Senegal)  Dakar (Senegal)  Dakar (Senegal)  Geradif (Sudan) | [14]  a  [4]  [16]  [17]  [18]  [19] | Sep to Dec 1988  Dec 1996 to Nov 2000  Dec 2000 to Apr 2005  Jan 1990 to Feb 1996  Oct 1997 to Mar 1999  Jul 1998 to Dec 1999  Oct 2000 to Dec 2001 | 282  340  1141  79  47  72  46 | 0-9  0-15  0-14  0-15  0-14  0-15  0-14 |
| Marked seasonality and 10-100pppy | Ouagadougou (Burkina Faso)  Maputo (Mozambique)  Same (Tanzania)  Macha (Zambia) | [20]  [22]  [13]  b | Jun to Oct 1993/1994  Feb 1995 to Apr 1997  Feb 2002 to Feb 2003  Jan 2003 to Dec 2004 | 204  189  59  280 | 0-15  0-6  0-13  0-5 |
| Marked seasonality and >100pppy | Sorou and Nayala (Burkina Faso) | [20] | Jun to Oct 1996 | 97 | 0-15 |
| **Respiratory distress** | | | | | |
| No marked seasonality and EIR<10pppy | - | - | - | - | - |
| No marked seasonality and EIR 10-100pppy | Libreville (Gabon)  Kumasi (Ghana)  Kilifi (Kenya)  Kilifi (Kenya)  Kilifi (Kenya)  Blantyre (Malawi) | [4]  [4]  [7]  [8]  [4]  [4] | Dec 2000 to Apr 2005  Dec 2000 to Apr 2005  Jul 1993 to Sep 1994  Sep 1999 to Dec 2000  Dec 2000 to Apr 2005  Dec 2000 to Apr 2005 | 139  1035  64  259  1071  331 | 0-14  0-14  0-13  0-14  0-14  0-14 |
| *Additional studies for sensitivity analysis^∫^* | HJH-Kinshasa (DRC)  PHK-Kinshasa (DRC)  Lambarene (Gabon)  Moshi (Tanzania) | [10]  [10]  [4]  [13] | Sep to Dec 2004  Sep to Dec 2004  Dec 2000 to Apr 2005  Feb 2002 to Feb 2003 | 6  5  38  28 | 0-12  0-12  0-14  0-13 |
| No marked seasonality and EIR >100pppy | Tamale (Ghana) | [27] | Aug 2002 to Nov 2002 | 66 | 0-9 |
| *Additional studies for sensitivity analysis^∫^* | Teule (Tanzania) | [13] | Feb 2002 to Feb 2003 | 20 | 0-13 |
| Marked seasonality and <10pppy | Banjul (Gambia, The)  Banjul (Gambia, The)  Dakar (Senegal) | a  [4]  [17] | Dec 1996 to Nov 2000  Dec 2000 to Apr 2005  Oct 1997 to Mar 1999 | 430  434  77 | 0-15  0-14  0-14 |
| Marked seasonality and 10-100pppy | Maputo (Mozambique) | [22] | Feb 1995 to Apr 1997 | 145 | 0-6 |
| *Additional studies for sensitivity analysis^∫^* | Same (Tanzania) | [13] | Feb 2002 to Feb 2003 | 12 | 0-13 |
| Marked seasonality and >100pppy | - | - | - | - | - |

^a^ M. Jallow, personal communication

^b^ P.E. Thuma, personal communication

*^∫^* Sites with less than 40 observations for a given outcome

**References**

1. Commey J: **Cerebral malaria in Accra, Ghana.** *Ghana Medical Journal* 1980, **19**:68-72.

2. Idro R, Karamagi C, Tumwine J: **Immediate outcome and prognostic factors for cerebral malaria among children admitted to Mulago Hospital, Uganda**. *Annals of Tropical Paediatrics: International Child Health* 2004, **24**:17-24(18).

3. Carme B, Bouquety JC, Plassart H: **Mortality and sequelae due to cerebral malaria in African children in Brazzaville, Congo**. *Am J Trop Med Hyg* 1993, **48**:216-221.

4. Taylor T, Olola C, Valim C, Agbenyega T, Kremsner P, Krishna S, Kwiatkowski D, Newton C, Missinou M, Pinder M, Wypij D: **Standardized data collection for multi-center clinical studies of severe malaria in African children: establishing the SMAC network**. *Trans R Soc Trop Med Hyg* 2006, **100**:615-622.

5. Steele RW, Baffoe-Bonnie B: **Cerebral malaria in children**. *Pediatric Infectious Disease Journal* 1995, **14**:281-285.

6. Snow RW, Armstrong Schellenberg JRM, Peshu N, Forster D, Newton CRJC, Winstanley PA, Mwangi I, Waruiru C, Warn PA, Newbold C, Marsh K: **Periodicity and space-time clustering of severe childhood malaria on the coast of Kenya**. *Trans R Soc Trop Med Hyg* 1993, **87**:386-390.

7. English M, Sauerwein R, Waruiru C, Mosobo M, Obiero J, Lowe B, Marsh K: **Acidosis in severe childhood malaria**. *Qjm* 1997, **90**:263-270.

8. Maitland K, Levin M, English M, Mithwani S, Peshu N, Marsh K, Newton CR: **Severe P. falciparum malaria in Kenyan children: evidence for hypovolaemia**. *Qjm* 2003, **96**:427-434.

9. Ikpatt NW, Asindi AA, Ekanem IA, Khalil MI: **Preliminary observations on cerebral malaria in Nigerian children**. *East Afr Med J* 1990, **67**:341-347.

10. Mulumba MP, Ilunga I, Bankoto A, Kamba VD: **Radioscopie du polymorphisme clinique de l'access de pernicieux palustre de l'enfant a Kinshasa**. *Congo Medical* Submitted

11. Koko J, Dufillot D, Zima-Ebeyard AM, Duong TH, Gahouma D, Kombila M: **Clinical and epidemiological aspects of malaria in children in Libreville, Gabon**. *Medecine d'Afrique Noire* 1999, **46**:10-14.

12. Bassat Q, Guinovart C, Sigauque B, Aide P, Sacarlal J, Nhampossa T, Bardaji A, Nhacolo A, Macete E, Mandomando I, Aponte J, Menendez C, Alonso P: **Malaria in rural Mozambique. Part II: children admitted to hospital**. *Malaria journal* 2008, **7**

13. Reyburn H, Mbatia R, Drakeley C, Carneiro I, Mwakasungula E, Mwerinde O, Saganda K, Shao J, Kitua A, Olomi R, Greenwood BM, Whitty CJ: **Overdiagnosis of malaria in patients with severe febrile illness in Tanzania: a prospective study**. *BMJ* 2004, **329**:1212.

14. Brewster DR, Kwiatkowski D, White NJ: **Neurological sequelae of cerebral malaria in children**. *Lancet* 1990, **336**:1039-1043.

15. Hensbroek MBv, Onyiorah E, Shabbar Jaffar, Schneider G, Palmer A, Frenkel J, Enwere G, Forck S, Nusmeijer A, Bennett S, Greenwood B, Kwiatkowski D: **A trial of artemether or quinine in children with cerebral malaria**. *New England Journal of Medicine* 1996, **335**:69-75.

16. Imbert P, Sartelet I, Rogier C, Ka S, Baujat G, Candito D: **Severe malaria among children in a low seasonal transmission area, Dakar, Senegal: influence of age on clinical presentation**. *Trans R Soc Trop Med Hyg* 1997, **91**:22-24.

17. Imbert P, Gerardin P, Rogier C, Ka AS, Jouvencel P, Brousse V, Guyon P: **Severe falciparum malaria in children: a comparative study of 1990 and 2000 WHO criteria for clinical presentation, prognosis and intensive care in Dakar, Senegal**. *Trans R Soc Trop Med Hyg* 2002, **96**:278-281.

18. Camara B, Diouf S, Diagne I, Fall L, Ba A, Ba M, Sow D, Kuakuvi N: **Severe malaria in children in a Senegal hospital setting.** *Médecine et Maladies Infectieuses* 2003, **33**:45-48.

19. Giha HA, El-Ghazali G, A-Elgadir TME, A-Elbasit IE, Eltahir EM, Baraka OZ, Khier MM, Adam I, Troye-Blomberg M, Theander TG, Elbashir MI: **Clinical pattern of severe Plasmodium falciparum malaria in Sudan in an area characterized by seasonal and unstable malaria transmission.** *Transactions of the Royal Society of Tropical Medicine and Hygiene* 2005, **99**:243-251.

20. Modiano D, Sirima BS, Sawadogo A, Sanou I, Pare J, Konate A, Pagnoni F: **Severe malaria in Burkina Faso: influence of age and transmission level on clinical presentation**. *American Journal of Tropical Medicine and Hygiene* 1998, **59**:539-542.

21. Schapira A, Solomon T, Julien M, Macome A, Parmar N, Ruas I, Simao F, Streat E, Betschart B: **Comparison of intramuscular and intravenous quinine for the treatment of severe and complicated malaria in children**. *Trans R Soc Trop Med Hyg* 1993, **87**:299-302.

22. Varandas L, Julien M, Gomes A, Rodrigues P, Van Lerberghe W, Malveiro F, Aguiar P, Kolsteren P, Van Der Stuyft P, Hilderbrand K, Labadarios D, Ferrinho P: **A randomised, double-blind, placebo-controlled clinical trial of vitamin A in severe malaria in hospitalised Mozambican children**. *Ann Trop Paediatr* 2001, **21**:211-222.

23. Schmutzhard E, Gerstenbrand F: **Cerebral malaria in Tanzania. Its epidemiology, clinical symptoms and neurological long term sequelae in the light of 66 cases**. *Trans R Soc Trop Med Hyg* 1984, **78**:351-353.

24. Slutsker L, Taylor TE, Wirima JJ, Steketee RW: **In-hospital morbidity and mortality due to malaria-associated severe anaemia in two areas of Malawi with different patterns of malaria infection**. *Trans R Soc Trop Med Hyg* 1994, **88**:548-551.

25. Schellenberg D, Menendez C, Aponte J, Guinovart C, Mshinda H, Tanner M, Alonso P: **The changing epidemiology of Malaria in Ifakara Town, southern Tanzania**. *Trop Med Int Health* 2004, **9**:68-76.

26. Olanrewaju WI, Johnson AWBR: **Malaria in children in Ilorin, Nigeria**. *East African Medical Journal* 2001, **78**:131-134.

27. Mockenhaupt FP, Ehrhardt S, Burkhardt J, Bosomtwe SY, Laryea S, Anemana SD, Otchwemah RN, Cramer JP, Dietz E, Gellert S, Bienzle U: **Manifestation and outcome of severe malaria in children in northern Ghana**. *Am J Trop Med Hyg* 2004, **71**:167-172.
